# Association of genetic variants at CETP, AGER, and CYP4F2 locus with the risk of atrophic age‐related macular degeneration

**DOI:** 10.1002/mgg3.1357

**Published:** 2020-07-14

**Authors:** Rasa Liutkeviciene, Alvita Vilkeviciute, Loresa Kriauciuniene, Mantas Banevicius, Brigita Budiene, Daiva Stanislovaitiene, Reda Zemaitiene, Vytenis P. Deltuva

**Affiliations:** ^1^ Neuroscience Institute Lithuanian University of Health Sciences Medical Academy Kaunas Lithuania; ^2^ Department of Ophthalmology Lithuanian University of Health Sciences Medical Academy Kaunas Lithuania

**Keywords:** age‐related macular degeneration, *CYP4F2*, *CETP*, *AGER*, *CYP4F2*, gene polymorphism

## Abstract

**Background:**

Age‐related macular degeneration (AMD) is the leading cause of blindness in the elderly individuals. The etiology of AMD includes environmental and genetic factors.

**Methods:**

We aimed to determine the association between *CETP* (rs5882; rs708272; rs3764261; rs1800775; rs2303790), *AGER* (rs1800624; rs1800625), and *CYP4F2* (rs1558139) gene polymorphisms and development of atrophic AMD. About 52 patients with atrophic AMD and 800 healthy control subjects were evaluated. The genotyping of single‐nucleotide polymorphisms in *CETP*, *AGER,* and *CYP4F2* was carried out using the real‐time‐PCR method.

**Results:**

Genetic risk models in the analysis of *CETP* rs5882 revealed statistically significant variables with increased risk of atrophic AMD in the codominant (*p* < .001), dominant (*p* < .001), recessive (*p* < .001), and additive (*p* < .001) models with the highest 25.4‐fold increased risk of atrophic AMD in the codominant model (*p* < .001). The *AGER* rs1800625 was associated with a highly increased risk of atrophic AMD in the codominant (*p* < .001), recessive (*p* < .001), and additive (*p* < .001) genetic models.

**Conclusion:**

We identified two polymorphisms with a higher risk of atrophic AMD (*CETP* rs5882 and *AGER* rs1800625).

## Graphical abstract

We aimed to determine the frequency of the genotypes of CETP(rs5882; rs708272; rs3764261; rs1800775; rs2303790), RAGE (rs1800624; rs1800625) and CYP4F2 (rs1558139) in patients (pts) with atrophic age‐related macular degeneration (AMD). We identified 2 polymorphisms with higher risk of atrophic AMD (rs5882 and rs1800625) and one protective polymorphism (rs3764261).

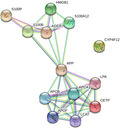

## 1 INTRODUCTION

Age‐related macular degeneration (AMD) is a common cause of blindness in developed countries and can progress from an early to intermediate, and finally, to the late forms, which can be atrophic or neovascular, and till this moment there is no effective treatment for geographic atrophy (Sallo et al., 2009). Early AMD involves the presence of small drusen and retinal pigmentary changes in macula; also there are two types of late AMD: dry AMD which is defined as geographic atrophy of the retinal pigmentary epithelium in the absence of neovascular AMD, and neovascular AMD including detachments of the retinal pigment epithelium (RPE), hemorrhages, and/or scars (Liutkevičienė, Čebatorienė, Liutkevičienė, Jašinskas, & Žaliūnienė, 2013). The prevalence of early AMD in persons 30–40 years of age is 1.3 ± 0.3%, and increases with age to 8.0% ± 5.5% in the ≥81‐year‐olds group (Nangia, Jonas, Kulkarni, & Matin, 2011). The risk of both late AMD forms increases with age and the prevalence reaches more than 10% in Europeans older than 80 years (Smith et al., 2001). Geographic atrophy (GA) occurs more commonly in individuals over 85 years of age (Klein et al., 2007). Europeans have a higher prevalence of geographic atrophy subtype (1.11%; 95% CI, 0.53%–2.08%) than Africans (0.14%, 0.04%–0.45%), Asians (0.21%, 0.04%–0.87%), and Hispanics (0.16%, 0.05%–0.46%) (Wong et al., 2014).

Geographic atrophy is a term used to describe dry AMD with the following clinical features: progressive atrophy of the RPE including processes of drusen formation (Wing, Blanchard, & Weiter, 1978). Sometimes geographic atrophy occurs after an RPE tear or rip and can be associated with ill‐defined or occult choroidal neovascular membranes (Meredith, Braley, & Aaberg, 1979).

It is known that AMD development is determined by many factors, but genetic factors play a significant role in AMD development (Fourgeux et al., 2012). Overall, the formation of drusen, local inflammation, and neovascularization are the most important pathogenic mechanisms causing the development of AMD. Aging has an impact on the accumulation of protein and oxidized cholesterol particles in the RPE and Bruch's membrane, known as drusen (Abu‐Asab, Salazar, Tuo, & Chan, 2013). Curcio, Millican, Bailey, and Kruth (2001) have found that these lipoproteins consisted of esterified cholesterol, which was sevenfold higher in the macula than in the periphery. Lipoproteins consist of protein components and lipids and are synthesized mainly in the liver. In Bruch's membrane, plasma is the main source of accumulated lipoproteins (Curcio et al., 2001). The accumulation of oxidized cholesterol is very important to AMD pathogenesis because the long‐term increase of high‐density lipoprotein (HDL) levels disrupts the function of HDL and promotes the formation pro‐oxidative and pro‐inflammatory particles, which leads to the reduced excretion of cholesterol and stimulates HDL oxidation (Kunchithapautham, Atkinson, & Rohrer, 2014). The oxidation products, such as lipid peroxides, bind lipids and form the outer wall of the basement membrane of the RPE forming drusen, causing inflammation and pathological angiogenesis (Ebrahimi & Handa, 2011). Impaired transport of substances between the choroidal capillaries and RPE disturbs the retinal function (Fliesler & Bretillon, 2010). Until now AMD etiology remains poorly understood, especially the geographic atrophy form. During the last few years, genetic research into AMD has been widely studied, but most studies have been analyzing exudative AMD form, and there are very few studies analyzing gene polymorphisms’ part in lipid metabolism association with atrophic AMD development (Fourgeux et al., 2012; Reynolds, Rosner, & Seddon, 2010; Tian, 2012). That is why in our study, we paid attention to the association of genes taking part in lipid metabolism with atrophic AMD. Only several studies have supported the association of increased risk of AMD development with variants of genes coding cholesterol ester transferase gene (*CETP*) (OMIM *118470) (Cougnard‐Grégoire et al., 2014; Liu et al., 2014; Wang, Han, et al., 2015). There was no positive association between AMD and Cytochromes P450 family (CYPs) (Fourgeux et al., 2012; Sakiene, 2016), and none of the studies have analyzed the association between single nucleotide polymorphisms of the receptor for AGE (*AGER*) (OMIM *600214) and any type of AMD. Another study has analyzed cholesterol‐24S‐hydroxylase (*CYP46A1*) (OMIM *604087) association with atrophic AMD and proved that *CYP46A1* rs754203 polymorphism per se is not associated with AMD (Fourgeux et al., 2012). Hageman et al. (2001) have proposed a hypothesis that inflammation and other factors of the immune response may play an important role in the stimulation of drusen formation and development of AMD as well. In recent years, the blockage of the neovascularization chain has been considered to inhibit the development of AMD. The vascular endothelial growth factor and the fibroblast growth factor are believed to promote angiogenesis, while it is inhibited by the pigment epithelial factor, angiostatin, endostatin, and others (Lee, Schloss, & Swain, 2000).

There are very few studies analyzing gene polymorphisms’ association with atrophic AMD, and results are inconsistent, so we aimed to shed light on the possible involvement of *CETP* (rs5882, rs708272, rs3764261, rs1800775, rs2303790), *AGER* (rs1800624 and rs1800625), and *CYP4F2* (OMIM *604426) (rs1558139) in atrophic AMD.

## 2 MATERIALS AND METHODS

### 2.1 Ethical compliance

The study was approved by the Ethics Committee for Biomedical Research at Lithuanian University of Health Sciences (LUHS) (Number—BE‐2‐/13). All subjects provided written informed consent in accordance with the Declaration of Helsinki. The study was conducted in the Department of Ophthalmology, Hospital of LUHS.

#### 2.1.1 Methods

The study groups were made of subjects who were admitted to the Hospital of Lithuanian University of Health Sciences Ophthalmology Department for preventive ophthalmological evaluation. A total of 852 subjects were evaluated, including 52 patients with geographic atrophy AMD, and 800 healthy controls. To ensure a sufficient number of subjects (patients with atrophic AMD and healthy controls), “Sample size calculation” formula was used in our research. It was calculated that 15 patients with atrophic AMD and 173 healthy controls must be included in our research.

#### 2.1.2 Control group formation

The control group consisted of subjects who had no ophthalmologic pathology on examination and who agreed to take part in this study. The inclusion criteria for control subjects were: (a) no ophthalmological eye disorders found on detailed ophthalmological evaluation, (b) detailed general clinical examination of the patients, and (c) participation consent. The exclusion criteria for control subjects were (a) any eye disorders and (b) any general therapeutic disorders.

#### 2.1.3 Ophthalmological evaluation

All study subjects were evaluated by slit‐lamp biomicroscopy to assess corneal and lenticular transparency. Classification and grading of lens opacities were performed according to the Lens Opacities Classification System III. At each examination, intraocular pressure was measured. Pupils were dilated with tropicamide 1%, after which fundoscopy, using a direct monocular ophthalmoscope, and slit‐lamp biomicroscopy with a double aspheric lens of +78 diopters, were performed. Results of eye examinations were recorded on special standardized forms. For a detailed analysis of the macula, stereoscopic color fundus photographs of the macula, centered at 45º and 30º to the fovea, were obtained with a Visucam NM Digital camera (Carl Zeiss Meditec AG).

The classification system of AMD formulated by the Age‐Related Eye Disease Study (“The Age‐Related Eye Disease Study system for classifying AMD from stereoscopic color fundus photographs: the Age‐Related Eye Disease Study Report Number 6,” 2001) was used: early AMD consisted of a combination of multiple small and several intermediate (63–124 μm in diameter) drusen, or retinal pigment epithelial abnormalities; intermediate AMD was characterized by the presence of extensive intermediate drusen and at least one large (≥125 μm in diameter) druse, or geographic atrophy not involving the center of the fovea; and advanced AMD was characterized by GA involving the fovea and/or any of the features of neovascular AMD (“The Age‐Related Eye Disease Study system for classifying AMD from stereoscopic color fundus photographs: the Age‐Related Eye Disease Study Report Number 6,” 2001).

The following subject exclusion criteria were used: (a) unrelated eye disorders, for example, high refractive error, cloudy cornea, keratitis, acute or chronic uveitis, diseases of the optic nerve; (b) systemic illnesses, for example, diabetes mellitus or conditions following organ or tissue transplantation; (c) ungraded color fundus photographs resulting from obscuration of the ocular optic system or because of fundus photograph quality. Information about general health was obtained from a family doctor examination and the data extract from medical documentation. Patients with atrophic AMD and control group subjects without pathologies mentioned above were included in our research.

#### 2.1.4 Single‐nucleotide polymorphism selection

Variants of *CETP, CYP4F2,* and *AGER* were chosen from the literature according to the mentioned associations with the pathological processes involved in AMD pathogenesis. Studies report the associations between *CETP* rs5882 and HDL‐C levels (Emamian et al., 2015) and Alzheimer‘s disease (Chen, Li, Zou, & Fu, 2014), which share the same pathological pathways as AMD (Xu, Cao, Rajapakse, & Matsubara, 2018). *CETP* rs708272 was shown to be associated with total cholesterol level changes (Iwanicka et al., 2018), with the risk of coronary atherosclerosis (Wang et al., 2013) and myocardial infarction (Semaev et al., 2019), which are linked to AMD as well (Liutkeviciene et al., 2012). *CETP* rs3764261 was also associated with serum HDL and APOA1 levels (Huang et al., 2019; Xu, 2019). Moreover, controversial results were found in AMD analysis, the minor allele at CETP rs3764261 was proved to be a risk factor to the development of AMD (Cheng, 2015), while in the other study it played the protective role (Wang, Zhou, et al., 2015). It was reported that *CETP* rs1800775 may influence serum HDL‐C levels as well (Huang et al., 2019; Wang et al., 2013), and the *CETP* rs2303790 variant was even strongly linked to increased risk of AMD development suggesting the possible genetic marker for AMD susceptibility (Cheng, 2015). *AGER* promoter polymorphisms (rs1800624 rs1800625) were also associated with cardiovascular disease (Buraczynska, Zaluska, Buraczynska, Markowska‐Gosik, & Ksiazek, 2015; Yu, 2013) and the *CYP4F2* rs1558139 was found to be associated with hypertension (Geng, Li, Wang, & Wang, 2019), which is one of the risk factors for AMD development as well as lipid metabolism changes mentioned above (Hyman & Neborsky, 2002).

#### 2.1.5 Protein–protein interactions

To evaluate the protein–protein interactions (PPIs) between these three genes, we used the STRING v11.0 tool (Szklarczyk et al., 2019). The STRING resource is available online at https://string‐db.org/.

Protein interactions in this database are divided into three main categories:

- Known interactions (experimental data (purple), curation in databases (blue)),
- Predicted interactions (neighborhood (green), gene fusion (red), co‐occurrence (dark blue), and
- Others (co‐expression (black), text mining (lime), homology (cyan)).

**Figure 1:**
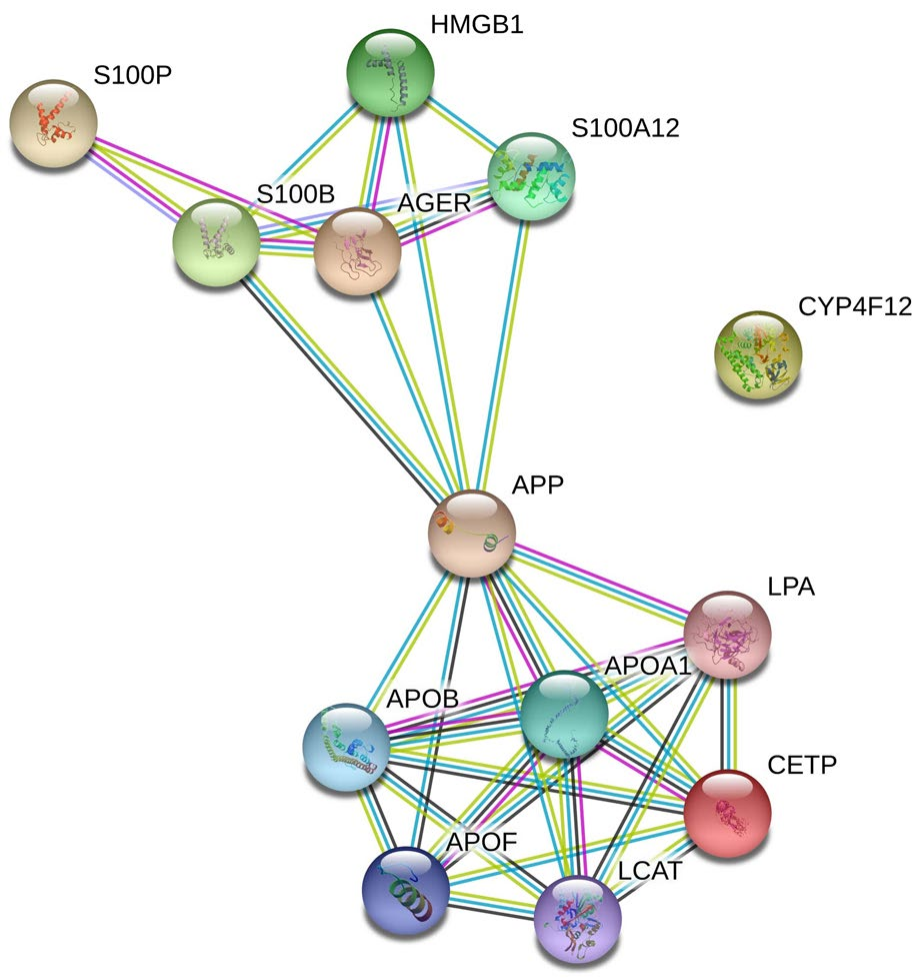
PPI network generated for CETP, AGER, and CYP4F2. No more than 10 interactions were displayed. Types of interaction sources include coexpression (black), experimental data (purple), curation in databases (blue), and text mining (lime). PPI enrichment *p*‐value = 4.63e‐07. PPI, protein–protein interaction

Protein interactions are determined and confirmed by genomic context, high‐throughput experiments, co‐expression, and previous publications in PubMed. PPI network for *CETP*, *AGER,* and *CYP4F2* was generated by default to connect these three genes with a minimum number of other genes into a network. A combined score of ≥0.4 for the nodes was considered to indicate a significant PPI interaction. Only interactions between *CETP* and *AGER* were determined using this tool (Figure 1).

#### 2.1.6 DNA extraction and genotyping

The genotyping of *CETP* (rs5882, rs708272, rs3764261, rs1800775, rs2303790), *RAGE* (rs1800624 and rs1800625), and *CYP4F2* (rs1558139) was carried out using the real‐time PCR. All single‐nucleotide polymorphisms (SNPs) were determined using TaqMan^®^ Genotyping assays (Thermo Scientific).

The genotyping was performed using a Rotor–Gene Q real‐time PCR quantification system (Qiagen). Appropriate real‐time PCR mixtures of *CETP* (rs5882, rs708272, rs3764261, rs1800775, rs2303790), *AGER* (rs1800624 and rs1800625), and *CYP4F2* (rs1558139) were prepared for determining SNPs.

A PCR reaction mixture (9 μl) was poured into each of 72 wells of the Rotor‐Disc, and then, 1 μl of matrix DNA of the samples (~10 ng) and 1 μl of a negative control (−K) were added.

The Allelic Discrimination program was used during the real‐time PCR. Then, the assay was continued following the manual provided by the manufacturer (www.qiagen.com, Allelic Discrimination). The program determined the individual genotypes according to the fluorescence intensity rate of different detectors: molecular marker labeled with VIC fluorescent dye was chosen for the X‐axis and a molecular marker labeled with FAM fluorescent dye was selected for the Y‐axis. These dye‐labeled probes were included in the TaqMan^®^ Genotyping assays.

#### 2.1.7 Statistical analysis

Statistical analysis was performed using the SPSS/W 20.0 software (Statistical Package for the Social Sciences for Windows, Inc.). Data are presented as absolute numbers with percentages in brackets and mean of age. Frequencies of genotypes (in percentage) are presented in Table 2. Differences were considered statistically significant when *p* < .05.

Hardy–Weinberg equilibrium (HWE) analysis was performed to compare the observed and expected frequencies of *CETP* (rs5882, rs708272, rs3764261, rs1800775, rs2303790), *RAGE* (rs1800624 and rs1800625), and CYP4F2 (rs1558139) using the chi‐square test in all groups. Analysis of HWE revealed that SNPs: *CETP* rs2303790 and *CETP* rs1800775 deviated from HWE (data not shown) and were excluded from the further statistical analysis. The distributions of the *CETP* (rs5882, rs708272, rs3764261), *AGER* (rs1800624 and rs1800625), and *CYP4F2* (rs1558139) SNPs in the AMD patient with geographic atrophy and control groups were compared using the chi‐square test or the Fisher exact test. Association of atrophic AMD with gene polymorphisms was quantified by Logistic regression analyses after controlling for age. Adjustment for age as adjusted odds ratios (OR) and its 95% confidence interval (95% CI) are presented in Table 3. The selection of the best genetic model was based on the Akaike Information Criterion (AIC); therefore, the best genetic models were those with the lowest AIC values. The Bioinformatics Institute's Online Sample Size Estimator (OSSE) was used to compute the statistical power (http://osse.bii.a‐star.edu.sg/calculation2.php). To reduce the possibility of a type I error due to multiple testing, Bonferroni correction and a *p* < .05/6 (since we analyzed six different SNPs after HWE testing) was applied.

## 3 RESULTS

The control group involved 800 subjects matched with the atrophic AMD patient group by gender. Females accounted for 63.5% in the group of patients and the control group (*p* = .347) (Table 1). Evaluating the age of all study subjects there was a statistically significant difference between patient and control groups: atrophic geography AMD patients were older than subjects in the control group (77.75 vs. 61.48, *p* < .001).

**Table 1:** Demographic characteristics of the study population

Characteristic	Group		*p* value
	Atrophic geography AMD *n* = 52	Control group *n* = 800	
Men, *n* (%)	17 (32.70	292 (36.5)	.347
Women, *n* (%)	35 (67.3)	508 (63.5)	
Age ± *SD*,	77.78 (9.8)	61.48 (11.3)	<.001

Abbreviation: AMD, age‐related macular degeneration.

Genotype and allele distribution analysis was performed as well (Table 2). Results showed that genotypes of rs5882 in the *CETP* gene had a statistically different distribution between patients and controls (*p* = .015) (Table 2). Further analysis revealed that genotypes of another SNP (rs3764261) in the *CETP* gene had a statistically different distribution, as well (*p* = .01) (Table 2). The analysis showed that allele A at *CETP* rs3764261 was significantly less frequent in atrophic AMD patients than in healthy controls (19.2% vs. 28.8%, *p* = .042) (Table 2). Allele distribution of rs1800625 in *AGER* gene was revealed to be significantly different comparing the atrophic AMD patient and control groups: G allele was statistically more frequently observed in atrophic AMD patients than in controls (22.1% vs. 14.6%, *p* = .047) (Table 2) and may be considered as a risk factor for atrophic AMD.

**Table 2:** Frequency of the genotypes and alleles of rs5882, rs708272, rs3764261, rs1800624, rs1800625, and rs1558139 polymorphisms in the patient with atrophic macular degeneration (AMD) and the control groups

Gene marker	Genotype/allele	Control *n* (%) (*n* = 800)	Atrophic AMD *n* (%) (*n* = 52)	*p* value*	Statistical power (%)
*CETP* rs5882	AA	380 (47.5)	32 (61.5)	.015	8.9
	AG	344 (43.0)	12 (23.1)		
	GG	76 (9.5)	8 (15.4)		
	A	1,104 (69.0)	64 (73.1)		
	G	496 (31.0)	28 (26.9)	1.0	
*CETP* rs708272	GG	243 (30.4)	17 (32.7)	.739	8.1
	GA	416 (52.0)	28 (53.8)		
	AA	141 (17.6)	7 (13.5)		
	G	930 (56.4)	62 (59.6)		
	A	698 (43.6)	42 (40.4)	.683	
*CETP* rs3764261	CC	409 (51.1)	37 (71.2)	.01	37.2
	CA	321 (40.1)	10 (19.2)		
	AA	70 (8.8)	5 (9.6)		
	C	1,139 (71.2)	84 (80.8)		
	A	461 (28.8)	20 (19.2)	.042	
*AGER* rs1800624	AA	320 (40.0)	20 (38.5)	.826	2.5
	AT	353 (44.1)	25 (48.1)		
	TT	127 (15.9)	7 (13.5)		
	A	993 (62.1)	65 (62.5)		
	T	607 (37.9)	39 (37.5)	1.0	
*AGER* rs1800625	AA	580 (72.5)	31 (59.6)	.109	24.1
	AG	206 (25.8)	19 (36.5)		
	GG	14 (1.8)	2 (3.8)		
	A	1,366 (85.4)	81 (77.9)		
	G	234 (14.6)	23 (22.1)	.047	
*CYP4F2* rs1558139	GG	235 (29.4)	17 (32.7)	.733	8.1
	GA	375 (46.9)	25 (48.1)		
	AA	190 (23.8)	10 (19.2)		
	G	845 (52.8)	59 (56.7)		
	A	755 (47.2)	45 (43.3)	.478	

Note

*CETP* GenBank Ref Seq, NC_000016.10.

*AGER* GenBank Ref Seq, NC_000006.12.

*CYP4F2* GenBank Ref Seq, NC_000019.10.

Abbreviation: AMD, age‐related macular degeneration.

* Bonferroni‐corrected significance threshold *p* = .05/6.

Genotype and allele distributions of *CETP* rs708272, *AGER* rs1800624, and *CYP4F2* rs1558139 did not differ between atrophic AMD patients and controls (Table 2).

Unfortunately, these results did not survive after Bonferroni correction (*p* > .05/6).

Binomial logistic regression analysis was performed to evaluate SNPs as the risk factors for atrophic AMD development (Table 3). Genetic risk models in analysis of *CETP* rs5882 revealed statistically significant variables with increased risk of atrophic AMD in the codominant (OR = 25.431, 95% CI: 9.960–64.932; *p* < .001), dominant (OR = 6.257; 95% CI: 2.737–14.306; *p* < .001), recessive (OR = 17.640; 95% CI: 8.430–36.909; *p* < .001), and additive (OR = 5.901; 95% CI: 3.547–9.815; *p* < .001) models with the highest 25.4‐fold increased risk of atrophic AMD in the codominant model (*p* < .001) (Table 3).

**Table 3:** Binomial logistic regression analysis of rs5882 and rs1800625 polymorphisms in the patient with atrophic age‐related macular degeneration and the control groups

Model	Genotype	OR (95% CI)^a^	*p* *	AIC
*CETP* rs5882				
Codominant	A/G	2.020 (0.762–5.351)	.157	231.297
	G/G	25.431 (9.960–64.932)	<.001	
Dominant	A/G + G/G	6.257 (2.737–14.306)	<.001	270.547
Recessive	G/G	17.640 (8.430–36.909)	<.001	231.333
*AGER* rs1800625				
Codominant	A/G	0 (‐)	.994	221.369
	G/G	22.553 (8.643–58.847)	<.001	
Recessive	G/G	31.891 (12.438–81.768)	<.001	240.203
Additive	—	2.645 (1.692–4.136)	<.001	277.346

Note

*CETP* GenBank Ref Seq, NC_000016.10.

*AGER* GenBank Ref Seq, NC_000006.12.

Abbreviations: AIC, Akaike Information Criterion; confidence interval.

^a^ OR—adjusted odds ratio by age.

* Bonferroni‐corrected significance threshold *p* = .05/6

Another SNP with revealed statistically significant association was *AGER* rs1800625 which was associated with highly increased risk of atrophic AMD in the codominant (OR = 22.553; 95% CI: 8.643–58.847; *p* < .001), recessive (OR = 31.891; 95% CI: 12.438–81.768; *p* < .001), and additive (OR = 2.645; 95% CI: 1.692–4.136; *p* < .001) genetic models (Table 3).

Binomial logistic analysis of *CETP* rs708272, *CETP* rs3764261, *AGER* rs1800624, and *CYP4F2* rs1558139 did not reveal any statistically significant variables (Table 3).

## 4 DISCUSSION

To our knowledge, only one study has investigated CYP (Fourgeux et al., 2012) gene polymorphism association with atrophic AMD, the other gene associations with atrophic AMD we analyzed for the first time. A study done by Fourgeux et al. (2012) have analyzed *CYP46A1* rs754203 polymorphism association with atrophic AMD and did not find any statistically significant differences between healthy controls and atrophic AMD patients. Sakiene (2016) study of *CYP4F2* (rs2108622) association with early and exudative AMD concluded that rs2108622 gene polymorphism had no predominant effect on the development of early AMD and exudative AMD. Stasiukonyte, Liutkeviciene, Vilkeviciute, Banevicius, and Kriauciuniene (2017) have analyzed two other SNPs at the *CYP2C19* (rs4244285) and *CYP1A2* (rs762551) genes and only rs762551 showed statistically significant results: Rs762551 C/C genotype was more frequently detected in patients with early AMD than in the control group (32.7% vs. 21.6%, *p* = .011) and was associated with an increased risk of developing early AMD (OR = 1.759, 95% CI: 1.133–2.729; *p* = .012). Considering SNPs of CYP family genes to be involved in AMD development, we analyzed rs1588139 at the *CYP4F2* gene association with atrophic AMD but results did not show any differences in genotype distribution or risk of atrophic AMD.

There are no studies analyzing the association between polymorphisms in the *CETP* gene and atrophic AMD form, while studies analyzing early and exudative AMD provide controversial results. We found only one study analyzing this *CETP* rs5882 gene polymorphism in the Chinese Han population by Zhang et al. (2013), which investigated exudative AMD patients and 204 controls, and *CETP* rs5882 was not associated with exudative AMD (Sobrin et al., 2011; Zhang et al., 2013). Studies analyzing *CETP* rs3764261 association with AMD are inconsistent, as well. One study found no association with AMD (Meng et al., 2015), another scientist group proved that this gene polymorphism can reduce AMD development (Wang, Zhou, et al., 2015), and three other reports proved statistically significant association with AMD development (Cougnard‐Grégoire et al., 2014; Liu et al., 2014; Wang, Han, et al., 2015).

To our knowledge, there is only one study analyzing *CETP* (rs1800775) gene polymorphism association with AMD in various populations (Restrepo et al., 2014). In this study, while *CETP* rs1800775 was associated with AMD in African Americans and Mexican Americans (*p* < .05), these associations did not survive strict corrections for multiple testing (Restrepo et al., 2014). Another SNP in the *CETP* gene (rs2303790) was analyzed in patients with exudative AMD and controls, showing a strong association between *CETP* and increased risk of AMD (Cheng, 2015). The *CETP* rs708272 gene polymorphism was analyzed in our study for the first time to our knowledge, and we cannot compare our results with other studies. After correction for multiple testing, genetic risk models in the analysis of rs5882 revealed statistically significant variables with increased risk of atrophic AMD in the codominant (*p* < .001), dominant (*p* < .001), recessive (*p* < .001), and additive (*p* < .001) models. Further analysis of rs3764261 did not survive this strict correction.

Analysis of two SNPs (rs2303790, rs1800775) in the *CETP* gene was not included in our study because these SNPs deviated from HWE.

Considering the involvement of SNPs in *AGER* gene in changes associated with atrophic AMD, we analyzed *AGER* gene and found rs1800625 was associated with a highly increased risk of atrophic AMD in the codominant (*p* < .001), recessive (*p* < .001), and additive (*p* < .001) genetic models and may be considered as a risk factor for atrophic AMD. Based on our results, we can conclude that rs1800625 in the *AGER* gene may impact the development of atrophic AMD. To our knowledge, there are no other studies analyzing *AGER* gene polymorphisms, so we can only compare our study with other experimental studies analyzing advanced glycation end products (AGEs) or its receptors (AGER). Schmidt, Du Yan, Yan, and Stern (2001), have found RAGE as a mediator affecting cellular activation, causing its dysfunction and following tissue destruction. Another study has found that accumulation of AGEs plays an important role not only in natural aging, but also in pathophysiological processes of human diseases and are pathogenic factors in visual disorders (Glenn & Stitt, 2009). Yamada et al. (2006) have found that upregulation of RAGE, AGER1, and AGER3 mRNAs in RPE cells overlying basal deposits (BDs) compared to RPE with normal Bruch's membrane, showed stronger immunohistochemical staining of RAGE and AGER1 in the RPE overlying BDs. These results confirm their earlier investigations, which revealed multiple changes caused by AGEs in the RPE–choroid, suggesting that AGEs start the whole cascade of processes in the aging mechanism as well as in age‐related diseases like AMD (Tian et al., 2005). RAGE is the AGE receptor involved in a variety of processes in multiple vascular and neural tissues and any changes in the *AGER* gene can alter receptor functional activity (Neeper et al., 1992).

Novelty. To our knowledge, *CYP4F2* rs1558139, *CETP* (rs5882, rs708272, rs3764261, rs1800775, rs2303790), *AGER* (rs1800624 and rs1800625) gene polymorphisms were evaluated in patients with atrophic AMD for the first time.

### 4.1 Limitations

We have a strong limitation of age differences between the study groups. The younger control group may not represent the general health conditions and the changes in eyes appearing in older age when atrophic AMD is determined because it is known that some diseases affect older people at higher rates. On the contrary, we included age as a covariate in logistic regression to control the age of study groups to avoid these differences and improve our results.

## 5 CONCLUSION

We identified two polymorphisms with a higher risk of atrophic AMD (*CETP* rs5882 and *AGER* rs1800625).

## CONFLICTS OF INTEREST

None of the authors have any proprietary interests or conflicts of interest related to this submission. This submission has not been previously published anywhere, and it is not simultaneously being considered for any other publication.

## AUTHOR CONTRIBUTIONS

Authors’ Contributions: RL, DS, BB, and RZ performed the Ophthalmological evaluation. AV and MB carried out the genotyping, AV carried out a part of genotyping, and performed the statistical analysis. RL drafted the manuscript. VPD participated in the design of the study and LK conceived of the study, and participated in its design and coordination and helped to draft the manuscript. All authors read and approved the final manuscript.

## Data Availability

The data that support the findings of this study are available on request from the corresponding author. The data are not publicly available due to privacy or ethical restrictions.
